# Hamstring stretch reflex: could it be a reproducible objective measure of functional knee stability?”

**DOI:** 10.1186/s40634-016-0040-x

**Published:** 2016-01-29

**Authors:** Jawad F. Abulhasan, Cameron M. Anley, Martyn D. Snow, Michael J. Grey

**Affiliations:** School of Sport, Exercise and Rehabilitation Sciences, University of Birmingham, Edgbaston Campus, Birmingham, West Midlands B15 2TT UK; The Royal Orthopaedic Hospital, The Woodlands, Bristol Rd South, Birmingham, West Midlands B31 2AP UK

**Keywords:** Anterior Cruciate Ligament ACL, Hamstring Activity, Short-latency response, Instability

## Abstract

**Background:**

The anterior cruciate ligament (ACL) plays an important role in anterior knee stability by preventing anterior translation of the tibia on the femur. Rapid translation of the tibia with respect to the femur produces an ACL-hamstring stretch reflex which may provide an object measure of neuromuscular function following ACL injury or reconstruction. The aim of this study was to determine if the ACL-hamstring stretch reflex could be reliably and consistently obtained using the KT-2000 arthrometer.

**Methods:**

A KT-2000 arthrometer was used to translate the tibia on the femur while recording the electromyography over the biceps femoris muscle in 20 participants, all with intact ACLs. In addition, a sub-group comprising 4 patients undergoing a knee arthroscopy for meniscal pathology, were tested before and after anaesthetic and with direct traction on the ACL during arthroscopy. The remaining 16 participants underwent testing to elicit the reflex using the KT-2000 only.

**Results:**

A total number of 182 trials were performed from which 70 trials elicited stretch reflex (38.5 %). The mean onset latency of the hamstring stretch reflexes was 58.9 ± 17.9 ms. The average pull force was 195 ± 47 N, stretch velocity 48 ± 35 mm/s and rate of force 19.7 ± 6.4 N/s.

**Conclusions:**

Based on these results, we concluded that the response rate of the anterior cruciate ligament-hamstring reflex is too low for it to be reliably used in a clinical setting, and thus would have limited value in assessing the return of neuromuscular function following ACL injuries.

## Background

The goal of rehabilitation following knee injury is to restore stability and function, allowing patients to return to their maximum level of function (Gregory et al. 2006; Bizzini and Dvorak 2015). The return to normal activity following injury is usually determined by an amalgamation of medical history, examination, combined with subjective physical assessment (Malanga et al. 2003) and sometimes instrumented testing devices and/or imaging (Campuzano Marín and Gómez-Castresana Bachiller 2010).

Numerous protocols have been advocated for return to sport participation following knee injury; however, there remains a lack of consensus in the literature as to the appropriate criteria on which to base this decision. In a recent review, Ardern et al. (2014) concluded that following anterior cruciate ligament (ACL) reconstruction, 82 % of patients return to some type of sport participation, 63 % return to pre-injury level of participation, and only 44 % return to competitive sport, despite their observation that 90 % were assessed as having normal or near normal function based on knee stability and lower limb strength testing. This discrepancy between return to sport participation and clinical findings highlights the possibility that additional factors that may not be assessed could be contributing to rehabilitation efficacy.

Ideally, neuromuscular function should be assessed in addition to standard stability and function assessments. Few studies have been conducted to better understand the applicability of an assessment of proprioception in the return to play decision (Beard et al. 1993; Friemert et al. 2005a; Schoene et al. 2009; Tsuda et al. 2001). Schoene et al. (2009) noted that both the mechanical and functional (sensorimotor) components of the knee play a role in the subjective feeling of instability following ACL reconstruction. They defined functional instability as a feeling of instability due to muscular dysfunction, which is caused by impaired neuromuscular function. This highlights the important role that proprioception plays in regulating the function of the muscles surrounding the knee. Various authors have noted that deficits in proprioception, balance, strength and neuromuscular control may persist for months following an injury or surgery (Lam et al. 2009; Torg et al. 1976; Tsuda et al. 2001). Therefore, assessment of these factors may be crticial for the return to play decision. Assessing these components in clinical practice can be challenging. As a result there is a need for clinic-based objective measures of neuromuscular function to aid the return to play decision.

Whilst these researcher have suggested this reflex could be used as a measure of knee stability, it has not been implemented it as a clinical-related measure. To allow the reflex to be used widely in a clinical setting, it would need to be obtained reliably with commonly available instrumentation, the most common of which is the KT-2000 arthrometer. Therefore, the aim of the present study was to investigate the efficacy of the KT-2000 to evoke the ACL-hamstrings reflex, thus providing an objective measure of neuromuscular function in a clinical setting.

## Methods

Our study sample was comprised of two groups. Eighteen healthy male participants (mean age 30 ± 6.6 y, range 24–46 y) were recruited as a sample of convenience from the student population of the University of Birmingham. A subset of six of these participants were also tested with the Vicon motion analysis system to better elucidate the movement of the tibia with respect to the femur. In addition, a set of 6 participants (5 ♂, 1 ♀; mean age 39.5 ± 13.8 y, range 20–51 y) were recruited to the study from the Royal Orthopaedic Hospital Birmingham, UK. These participants were patients undergoing arthroscopic meniscectomy or diagnostic arthroscopy, all of whom had intact and healthy ACL. In all cases the participants were healthy adults with no history of knee instability. Participants were excluded from the study if they had any neurological condition or impairment of the involved limb, inability to give informed consent, history of ACL reconstruction, ligamentous instability, previous knee surgery or fracture, and clinical or radiological evidence of osteoarthritis.

All participants provided written informed consent and the study was conducted according to the Declaration of Helsinki. Ethical approval for the laboratory-based component of the study was obtained from the University of Birmingham Science, Technology, Engineering and Mathematics (STEM) Ethics Committee (ERN_13-0290) and approval for the clinical component was obtained from the Integrated Research Approval System (IRAS project ID 13-0164).

All trials were conducted by a physiotherapist (JA) experienced with the KT-2000 arthrometer (MEDmetric Corp, San Diego, CA, USA). Surface EMG (Biometrics, Ltd., Newport, UK) electrodes were placed on the belly of the biceps femoris muscle in accordance with SENIAM guidelines (Hermens et al. 2000). The position and force outputs from the KT-2000 were connected to the data acquisition system. These analogue traces were bandpass filtered at 20–500 Hz and sampled at 5 kHz. All data were stored on an encrypted computer for offline analysis.

In order to elicit a stretch reflex the force of the pull and subsequent strech velocity of the tibial translation must be sufficient to activate the proprioceptors. The velocity of the pulls was generated as fast as possible by the tester (JA). For the force magnitude, Friemert et al. (2005b) demonstrated that a force of ≥ 140 N and stretch velocity of 30 mm/s would increase the chance to elicit stretch reflex to 100 %. In a pilot experiment, the force of pull on the handle of the KT-2000 measured using a strain gauge. The force output of the KT-2000 was determined statically with calibration weights within the range of forces expected to be generated by the user. Similarly, the position output of the KT-2000 was calibrated using ceramic test gauge blocks.

The KT-2000 arthrometer was used with the Lachman test (Daniel et al. 1985) in which the assessor produces an anterior displacement of the tibia with respect to the femur. The participant remained relaxed in a supine position on the examination bed with the dominant knee supported at 30° of flexion. A goniometer was used to ensure the knee was placed in 30° flexion. The KT-2000 was secured over the participant’s leg in the ideal position with reference to the knee joint line. Both knees were supported on a firm comfortable platform placed proximal to the popliteal space. In addition, a foot support, was used to position the leg symmetrically and avoid external rotation of the tibia. Next, a Lachman test was performed by pulling rapidly on the handle of the KT-2000. Each participant underwent 3 trials, with 3 attempts in each trial to elicit hamstring stretch reflex. Furthermore, to determine the onset and magnitude of the tibial translation, a control experiment was performed with six participants using a high-speed 3D motion analysis system (Vicon MX Nexus system, Vicon Motion Systems Limited, Oxford Metrics Ltd., Oxford, UK). Retro-reflective markers were fixed to the skin using double-sided adhesive tape over the lateral femoral epicondyle, 3 cm above the lateral femoral epicondyle, 3 cm below the lateral tibial epicondyle and on the apex of the patellae. Three dimensional movement was capture by six cameras, calibrated with a residual error less than 1 mm and a sampling rate of 500 Hz.

**Fig. 1:**
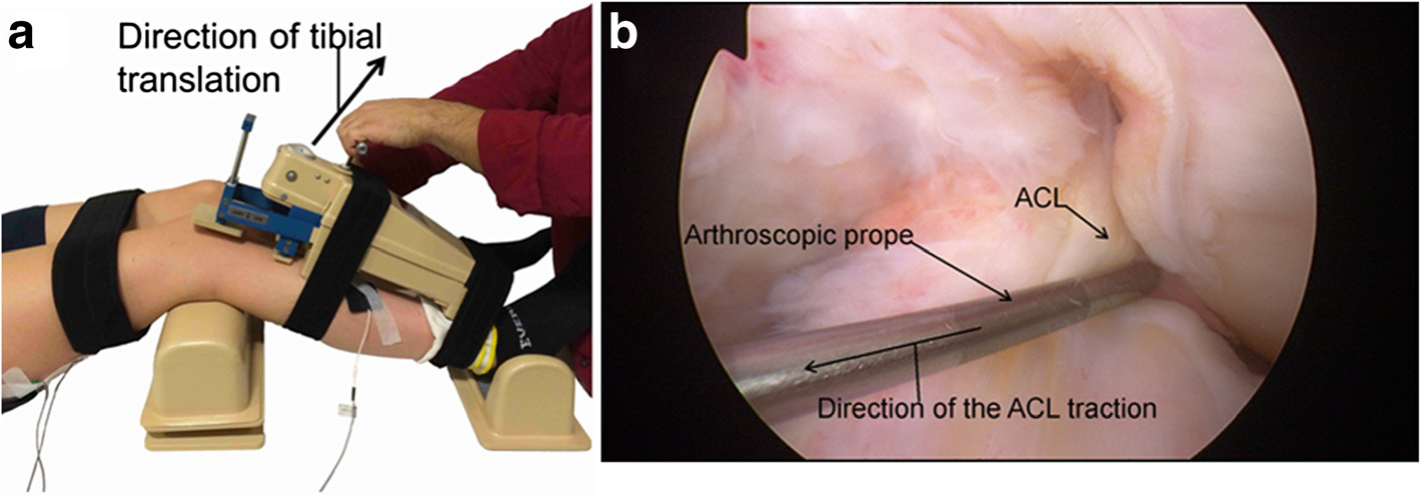
**a**. shows the expiremnetal procedure of the KT-2000 arthrometer and the position of the knee joint, placement of the examiner hands and the direction of the tibial pull illustrated. **b.** shows the position of the arthroscopic prope and the direction of the ACL pull by the examiner. The tibial translation with the KT-2000 and, by inference, the tendon movement is similar to that of the direct ACL pull reported by Friemert et al. (2005a). This does mean to imply that the reflex onset latency between the two methods is the same

In addition, to specifically confirm that the muscle activity traces were indeed the ACL/Hamstring reflex rather than movement artefacts, we compared the responses elicited with the KT-2000 to responses elicited from direct traction on the ACL during arthroscopy (Friemert et al. 2005a). In this control experiment, four participants scheduled for an arthroscopic meniscectomy were recruited to the study. Reflexes were initially elicited with the KT-2000 inside the operating theatre using a very similar procedure described by Friement et al. Whilst we could not specifically control the force from direct traction in the same manner, we did ensure the force was greater than that of Friemert et al. (2005a). Furthermore, precautions were taken to replicate the same procedure in our laboratory-based experiments: 1) the knee angle was maintained at 30°, 2) the placement of the EMG electrodes was standardized for all participants, 3) participants were placed in the same position, 4) the technique used for the pulling force was similar in the laboratory and arthroscopic procedures (Fig. 1). Reflexes were elicited initially with the patients awake and again following anaesthesia. Patients were then placed under general anaesthesia using propofol and sevoflurane, it is unlikely that the excitability of the monosynaptic reflex arc or neuromuscular function were affected by the dosage of drugs used (see Kerz et al. (2001). Sterile surface markers and leads were then placed over the hamstings in order to eliminate contamination of the operative site. All arthroscopies were performed by a single consultant orthopaedic surgeon (MS). During arthroscopy direct anterior traction was placed on the ACL using an arthroscopic probe, and the subsequent electromyographic activity measured within the hamstrings as described by Friemert et al. (2005a).

Mean ± SD of the stretch reflexes responses was calculated to produce a percentage of reflex across all participants. Morover, a *t*-test was excuted to assess the difference between the onset latencies of the laboratory groub and the sub-set of the camera group. All calculations were made using SPSS Statistics software Version 21 (IBM Corporation,New York, USA).

**Fig. 2:**
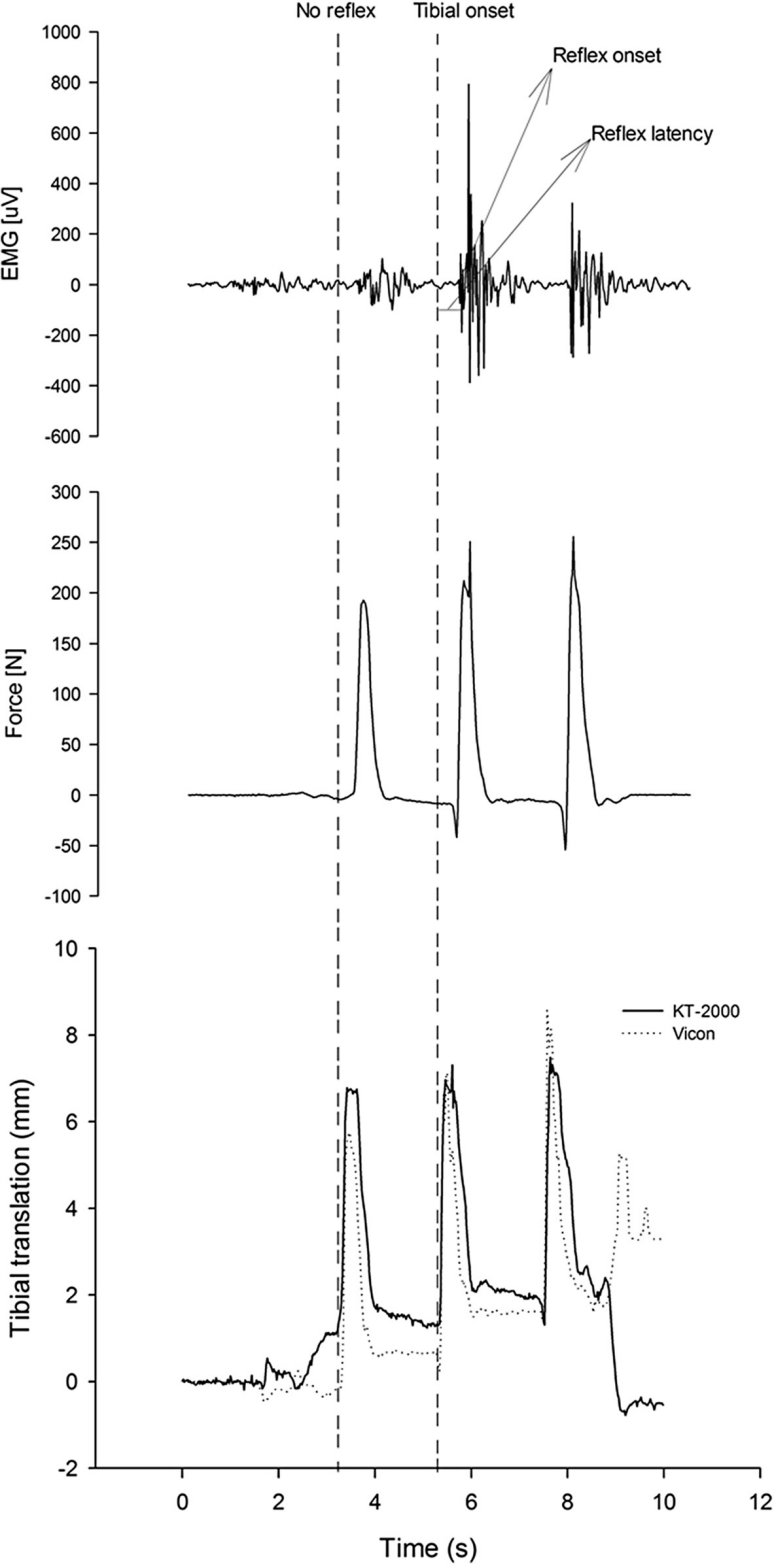
An example of a participant’s EMG recorded from the biceps femoris muscle which shows the responses obtained after a single trial with three attempts to elicit a reflex using the KT-2000. EMG, force and position measured with the KT-2000 and vicon system, respectively, are illustrated. The onset of tibial translation and onset latency of the reflex are illustrated. Reflex responses are observed with the second and third pulls, but not the first pull

The force of the pull was calculated for each trial. The presence of a stretch reflex in response a pull was calculated to produce a percentage of reflex across all participants. Reflex onset latency was determined from all traces with an reflex. detect the onset latency of the stretch reflex response, a time window was defined in a 20—50 ms after the stretch onset; which incorporates the physiological range for the onset of a reflex (Friemert et al. 2005b). Within this window, the onset of the reflex was determined by visual inspection comparing the magnitude of the response with the background EMG. The onset latency of the reflex was defined as the time delay from the onset of tibial translation to onset of the first major deflection in the reflex (see Fig. 2).

## Results

**Fig. 3:**
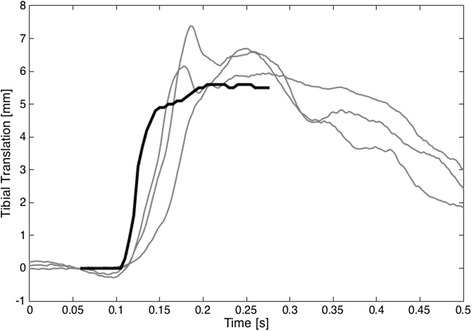
A comparison of the tibial translation acquired by direct tendon pull Friemert et al. (2005a) (black line) and the tibial translation from a single trial with three translations elicited with the KT-2000 (grey lines). The tibial translation (mm) induced with the KT-2000 and, by inference, the tendon movement is similar to that of the direct tendon pull reported by Friemert et al.(2005a). This does not mean to imply that the reflex onset latency between the two methods is the same. It is likely that the ACL does not take up the stretch immediately when the tibia is translated as this would explain the longer latency in our findings (58.9 ± 17.9) compared with Friemert et al. (2005a)

**Fig. 4:**
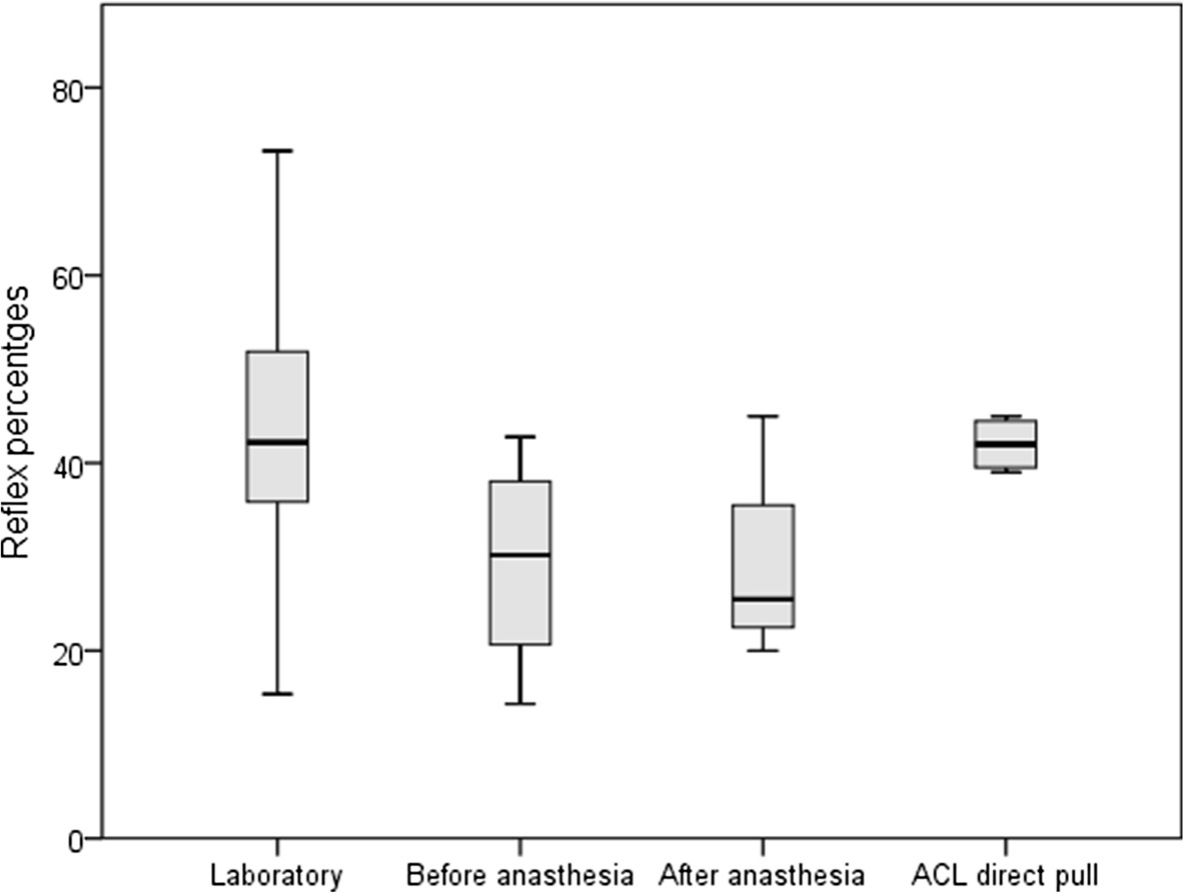
Box plot of hamstring stretch reflex percentages in all testing conditions. The laboratory group consists of 16 participants 38.5 % while the arthroscopic group consists of 4 participants; 30 % before anaesthesia, 29 % after anaesthesia and 42 % with a direct pull. The low percentages represents the lack of reproducibility of the hamstring stretch reflex when used as an objective measure of knee functional stability

Tibial translation (mm) with the KT-2000 is similar to that elicted when the tendon is directly pulled (Fig. 3). On average, the force (195 ± 47 N), stretch velocity (48 ± 35 mm/s) and rate of force (19.7 ± 6.4 N/s) of pull that elicited stretch reflexes in the present study were not significantly different from those perturbations that did not elicited stretch reflexes 202 ± 42 N (*p* = 0.32), 50 ± 28 mm/s (*p* = 0.76) and 18.3 ± 6.0 N/s (*p* = 0.16) respectively. On average, 38.5 % of the trials in the laboratory group elicited a stretch reflex. In the arthroscopy group, there was no difference in the percentage of reflexes elicited with the KT-2000 before anaesthesia (30 %) and after anaesthesia (29 %), or with direct traction on the ACL (42 %) (Fig. 4). The mean onset latency of the hamstring reflex across all trials was 58.9 ± 17.9 ms. The mean onset latency of the hamstring reflex laboratory group (61.7 ± 17.4 ms) and the sub-set of the camera group (58.7 ± 18.9 ms) were not statisticly different (*p* = 0.472). A correlation analysis reveals that age was not correlated with either the number of reflexes (r = 0.074) or the onset latency (r = 0.078).

## Discussion

The aim of this study was to determine if the ACL hamstring stretch reflex could provide a reproducible objective clinical measure to assess the neuromuscular functional stability of the knee. Hence, we aimed to quantify the robustness of ACL hamstring stretch reflex that could be generated using the KT-2000. The tibial translation (mm) induced with the KT-2000 and, by inference, the tendon movement is similar to that of the direct tendon pull reported by Friemert et al. (2005a). Whilst the force of the pull (195 ± 47 N), rate of force (19.7 ± 6.4 N/s) and the velocity of tibial translation (49 ± 35 mm/s) were sufficient to elicit a reflex, only 38.5 % of the trials elicited a stretch reflex. As a result, we must conclude that the ACL-hamstring stretch reflex elicited with the KT-2000 arthrometer is not sufficiently reproducible to provide a reliable clinical measure.

The anterior cruciate ligament-hamstring reflex has been proposed as an objective measure of the neuromuscular state of knee both arthroscopically (Friemert et al. 2005a) and clinically (Schoene et al. 2009). In both cases, the ACL-hamstring reflex was suggested to be clinically useful. Schoene et al.’s (2009) investigated the reliability of such technique on the bases of intra-individual reproducibility, inter-examiner reliability, fatigue, weight, height and physical fitness. Their protocol involved participants standing with the knees flexed at 30°, the feet externally rotated to 5° and the patella against a supporting plate. Posterior–anterior tibial translation is induced by a 300 N force applied via a pneumatic cylinder and piston, 10 cm below the popliteal fossa and parallel to the tibial plateau. Only the inter-tester reliability showed a significant difference between examiners. On the other hand, all the remaining factors showed no significant differences. This study concluded that none of the above-mentioned factors had a relevant influence on the reflex responses. Schoene et al. (2009) suggested that their method may assist in establishing a clinically relevant testing system for the functional stability of the knee. However, their protocol is fairly complex and requires specific equipment not commonly available in a clinical scenario.

The force of the perturbation in the present study was less that that reported by Friemert et al. (2005a) (300 N compared with approximately 200 N in the present study). However, in a pilot study undertaken on 10 participants, Friemert et al. (2005b) demonstrated that below 50 N no reliable response could be obtained, but the response rate increased to 80 % at 90 N and 100 % over 140 N. These results are similar to those shown by Jennings and Seedhom (1994), who used a 140 N force in their study as they could not demonstrate contraction of the hamstrings below 100 N. In line with their conclusion that the higher perturbation forces the higher chance of eliciting the stretch reflex, our method of eliciting the stretch reflex used the strongest and fastest perturbation possible The mean rate of force and stretch velocity in the present study was 19.7 ± 6.4 N/s and 49 ± 35 mm/s respectively, which exceeded the minimimum velocity (30 mm/s) recommended by Friemert et al. (2005b) to obtain a reliable reflex rersponse. On the conterary, Bedingham and Tatton (1984) reported that stretch reflex latency being independent of force when tested on Monkeys. However, consistency of our methodological procedure was maintained by trying to have the same experienced user perform the procedure through the study. Force, rate of force and stretch velocity was recorded and examined from all trials. No trials were eliminated for the possibility that the force was not strong enough. This is illustrated in Fig. 2 which showed an example of data of a single trial with the amount of force for each of the three pulls.

Although not reported in all studies, Tsuda et al. (2001) demonstrated that the elicited rate of the hamstring stretch reflex through a direct electrical stimulation of the ACL was 8/9 for the biceps femoris and 5/9 for the semitendinosis muscles. With direct traction on the ACL, Friemert et al. (2005b) demonstrated the median latency response aspect of the reflex 10/10 times in the medial hamstrings and 8/8 times in the lateral hamstrings. Unlike invasive studies, the specific rate of acquisition of stretch reflexes at non-invasive studies has not been reported (Schoene et al. 2009; Melnyk and Gollhofer 2007b; Friemert et al. 2010). This is however important when considering the reproducibility and applicability of such a method in clinical settings.

In a sub-group of our sample, the arthroscopy group, a battery of trials were performed after anaesthesia to exclude artefacts and confirm that the reflexes obtained were in fact related to the ACL-hamstring reflex. Although small, the reflexes in this group were seen as the gold standard against which the remainder of the tests were compared (30 ± 12 % before anaesthesia and 29 ± 11 % after anaesthesia, or 42 ± 3 % with direct traction on the ACL). The rate at which the stretch reflex was obtained was similar to the laboratory group. Therefore, in contrast with Friemert et al. (2005a), the inability to obtain consistent stretch reflex percentages during the four different situations shows why the hamstring stretch reflex cannot be used as an objective measure of knee functional stability. Although we tried to replicate Friemert et al.’s (2005a) work (Fig. 3), we could not reproduce their results in the arthroscopic tests of our study. Whilst they used a well defined perturbation with a force of 300 N to elicit stretch reflexes while we used the strongest and fastest possible manual perturbation done by the surgeon’s hand. Furthermore, we the manual technique using the KT-2000 produces a faster stretch velocity (49 ± 35 mm/s) and higher rate of force (19.7 ± 6.4 N/s) and this would increase the likelihood of eliciting stretch reflexes. Nonetheless, our results showed that the mean onset latency of the stretch reflexes were 58.9 ± 17.9 ms compared to (Friemert et al. 2005a) who reported 23.9 ± 1.7 ms. The tibial translation (mm) with the KT-2000 and, by inference, the tendon movement is similar to that of the direct ACL pull reported by Friemert et al. (2005a). It is likely very likely that the ACL does not take up the stretch immediately when the tibia is translated with respect to the femur. This would explain the longer latency in our findings compared with Friemert et al. (2005a) who used a direct pull on the tendon.

In the laboratory group, the rate of reflexes was slightly, but not significantly, higher (41 ± 11 %). Although a reflex was obtained in every patient (range of 44.9), the reflexes were not consistently elicited. Accordingly, in our study, we were unable to reproducibly elicit the ACL-hamstring reflex using the KT-2000 arthrometer. Based on the inconsistent nature of the reflex, we feel that the reflex elicited by the KT-2000 could not be used in clinical practice as a measure of knee functional stability.

The lack of standardisation of force used to induce tibial translation with the KT-2000 arthrometer is a weakness of our study. Although the lack of standardisation can be considered as a weakness, it does however represent the intra-individual variability that exists in human clinical-related practice, and is therefore relevant to the application of such techniques in clinical settings. In addition, we acknowledge that we assessed the reflex in a small number of intraoperative cases, which was however used only to confirm the characteristics of a gold standard reflex to be used as a reference standard for our laboratory testing. To minimise variability, all tests were by the same investigator, who was familiar with the KT-2000 arthrometer.

## Conclusion

Although it was possible to elicit the anterior cruciate ligament-hamstring stretch reflex with direct traction on the ACL intraoperatively and with the KT-2000 device in every participant in the present study, the reflexes could not be evoked consistently. We therefore conclude that the anterior cruciate ligament-hamstring stretch reflex cannot be elicited with the KT-2000 reliably in a clinical setting. It would therefore have limited value in assessing the return of neuromuscular function following ACL injury. Further studies need to be conducted to investigate other robust ways to objectively measure knee neuromuscular stability.

## Abbreviations

ACL: Anterior Cruciate Ligament
EMG: Electromyography
Ms: Milliseconds
SD: Standard Deviation
STEM: University of Birmingham Science, Technology, Engineering and Mathematics
IRAS: Integrated Research Approval System
PI: Principal Investigator
JA: Jawad Abualhasan
MS: Martyn Snow
CA: Cameron Anley
MG: Michael Grey
